# Endothelial, Vascular and Sympathetic Alterations as Therapeutic Targets in Chronic Heart Failure

**DOI:** 10.3390/biomedicines11030803

**Published:** 2023-03-06

**Authors:** Fosca Quarti-Trevano, Raffaella Dell’Oro, Cesare Cuspidi, Pasquale Ambrosino, Guido Grassi

**Affiliations:** 1 Clinica Medica, School of Medicine and Surgery, University Milano-Bicocca, 20052 Monza, Italy; 2 Istituti Clinici Scientifici Maugeri IRCCS, Directorate of Telese Terme Institute, 82037 Telese Terme, Italy

**Keywords:** chronic heart failure, endothelial function, sympathetic activity, cardiovascular drugs, physical training, rehabilitation, renal denervation, carotid baroreceptor stimulation

## Abstract

Vascular and sympathetic abnormalities characterize chronic heart failure (CHF). Alterations include (1) a reduction in arterial distensibility, (2) endothelial dysfunction, (3) a decrease in arterial compliance and a parallel increase in arterial stiffness, and (4) sympathetic cardiovascular activation. Altogether, these alterations represent important targets in therapeutic interventions, because they display an independent negative impact on the disease prognosis, favouring disease progression and the development of cardiovascular complications with direct and indirect mechanisms. The present review will examine the effects of the different therapeutic interventions targeting the vascular/sympathetic alterations detected in CHF. Non-pharmacological, pharmacological and device-based treatments will be discussed in detail, highlighting the possible mechanisms responsible for the vascular/sympathetic effects of each intervention. Finally, the unmet goals in treatment in relation to endothelial and adrenergic targets will be also discussed.

## 1. Introduction

Chronic heart failure (CHF) is characterized by significant alterations in vascular function that affect the visco-elastic properties of large, medium-size and small arteries, including microcirculation [1,2]. The pathophysiological and clinical relevance of these alterations, which also embrace endothelial dysfunction, is documented by evidence indicating that both arterial distensibility and vascular compliance play key roles in cardiovascular homeostatic control by modulating a number of important functional variables, such as arterial impedance, cardiac afterload and myocardial oxygen consumption [3]. In the case of CHF, the clinical relevance of vascular alterations is strengthened by the observation, reported in several prospective studies, that endothelial dysfunction, and arterial and arteriolar vascular alterations display adverse independent impacts on the disease prognosis, as they are directly and indirectly involved in the progression of CHF and in the development of cardiovascular complications [4,5,6]. Vascular alterations detectable in CHF occur as a result of complex interactions between static and dynamic changes involving several mechanisms regulating the structure as well as the function of the arterial wall. A leading factor is represented by sympathetic activation, which has been repeatedly documented to represent a key pathophysiological hallmark of the CHF state [7]. Indeed, the activation of sympathetic influences on the arterial wall has been shown to increase arterial stiffness, reduce vascular distensibility and promote the development and progression of endothelial dysfunction [6,7]. Furthermore, similarly to what has just been mentioned for vascular alterations, sympathetic abnormalities represent independent markers of an adverse disease prognosis [7]. Taken together, this evidence represents the pathophysiological background for considering sympathetic/vascular alterations to be important targets in therapeutic interventions aimed at improving the clinical course of disease, counteracting cardiovascular complications and ameliorating the quality of patients’ life.

**Figure 1:**
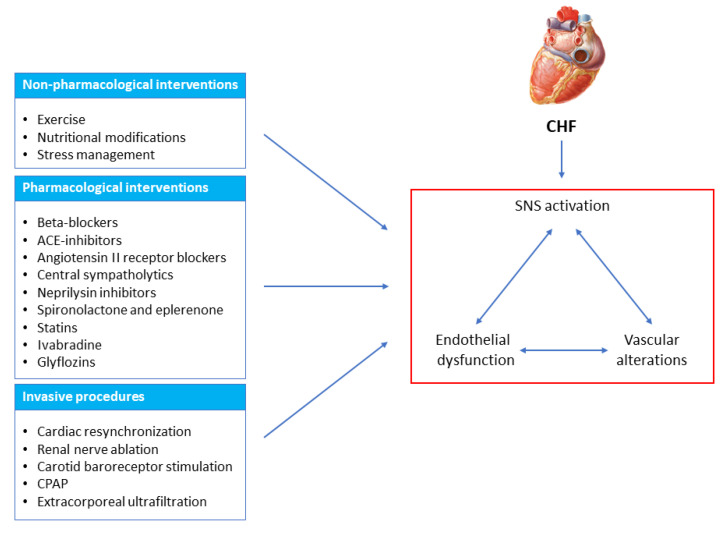
Scheme illustrating various non-pharmacological, pharmacological and device-based therapeutic interventions acting on the sympathetic/vascular/endothelial dysfunction characterizing chronic heart failure (CHF). SNS, sympathetic nervous system; CPAP, continuous positive airway pressure.

The present paper, after briefly recalling the methodological approaches to assess vascular, endothelial and sympathetic functions in human beings, will examine the effects of different therapeutic interventions targeting the vascular/sympathetic alterations characterizing CHF (Figure 1). This will be performed by reviewing non-pharmacological, pharmacological and invasive instrumental approaches currently employed in clinical settings. The goals of therapeutic interventions having vascular and sympathetic alterations as targets that have not yet been accomplished in current clinical practice will also be specifically mentioned. These unmet objectives will likely represent the goals of research in this area in the years to come.

## 2. Assessment of Vascular–Sympathetic Functions

A variety of methods have been developed throughout the years for examining vascular and endothelial functions at the levels of large, medium-size and small arteries in humans. Historically, among the first approaches employed, two methods are worthy of mention. The first one is based on the intra-arterial infusion of compounds causing endothelium-dependent vasodilation, such as acetylcholine, or triggering an increase in nitric oxide synthesis and release, such as nitroglycerine and nitroprusside. The resulting vasodilation, initially assessed using quantitative angiography [8], has been more precisely quantified over the years using intra-coronary Doppler, magnetic resonance or positron-emission tomography [9,10]. The second approach makes use of the plethysmographic technique and is based on the occlusion of the brachial artery of the dominant arm associated with dynamic exercise using the transient inflation of a sphygmomanometric bladder [11]. Arterial occlusion is rapidly removed, while venous occlusion is maintained, and the arterial flow increase (indicating vasodilation) is measured using a mercury strain gauge, which assesses the increased volume of the forearm, proportional to the arterial flow.

In more recent years, two other sophisticated, non-invasive approaches have been employed. The first one is based on a pulsed ultrasound wall tracking device, which allows one to assess pulse wave velocity; cross-sectional compliance; arterial distensibility; and diameter at the levels of the carotid, brachial and femoral arteries [12]. The other approach, complementary to the previous one, is peripheral arterial tonometry, which gives the possibility to assess endothelial function using sophisticated distal digit transducers [13]. The method is particularly useful in large-scale population studies, and its utility has been confirmed in the Framingham heart study [4].

As far as sympathetic functional assessment in humans is concerned, several approaches can be used. These include less or more sophisticated and specific methodologies. Among the first ones, mention should be made of the assay of neuroadrenergic transmitter norepinephrine in peripheral venous blood and of the power spectral analysis of heart rate signal in specific low- and high-frequency bands [7]. Much more sensitive approaches are (1) the microneurographic recording of efferent postganglionic sympathetic nerve traffic in the peroneal nerve [7], (2) the regional radiolabelled norepinephrine spillover approach [7] and (3) the neuroimaging techniques for visualizing cardiac sympathetic innervation [7]. All these techniques have specific advantages and limitations, a consideration that may emphasize the need for performing sympathetic evaluation based on the combination of two different approaches.

## 3. Non-Pharmacological Interventions

The vascular and sympathetic dysfunctions reported in CHF are favourably affected by the no-drug therapeutic interventions currently employed in the treatment of the disease. A considerable number of data, in particular, have been collected for three procedures, namely, physical exercise training, continuous positive airway pressure and extracorporeal ultrafiltration.

Since the seminal observation made by German investigators more than a quarter of century ago documenting the improvement in endothelium-mediated flow-dependent dilatation in a small group of CHF patients who underwent daily isometric handgrip exercise training during a 4-week period [14], several studies have confirmed the favourable effects of regular physical exercise on vascular function. This was particularly the case when the training programme consisted of dynamic exercise (bicycle and exercise bike training, walking, light running, etc.). Recently, a meta-analysis based on the data collected in 18 studies enrolling, overall, more than 800 patients with CHF has confirmed the improvement in flow-mediated vasodilation as an index of arterial stiffness; the patients followed a physical training programme of variable intensity for a period of time lasting between 4 and 12 weeks according to the study protocols [15]. Of special interest were the observations that (1) there was a significant direct relationship between the magnitude of vascular improvement and the intensity of the physical training programme [16] and (2) the favourable vascular effects of a regular exercise programme were similarly observed in CHF with reduced or preserved left ventricular ejection fraction [15]. Among the various mechanisms proposed throughout the years for explaining the improvement in vascular and endothelial functions induced by exercise training, a leading one is represented by the modulatory effects of the intervention on the sympathetic activation (and thus on sympathetic vasoconstriction) characterizing the disease. Indeed, regular physical exercise may not only improve the New York Heart Association (NYHA) functional class, systemic haemodynamics and exercise capability but also exert profound sympathoinhibitory effects [16]. These have been documented using different methodological approaches to assess sympathetic function, including the power spectral analysis of heart rate signal, the systemic norepinephrine spillover technique and the direct recording of efferent postganglionic muscle sympathetic nerve activity in the peroneal nerve, with a decrease in the mean values amounting to 40% [16]. In a meta-analysis of recent publications, the effects of exercise training rehabilitation on sympathetic neural drive were evaluated in CHF patients with reduced left ventricular ejection fraction [17]. The results show that the procedure allows important clinical benefits (amelioration of functional class, symptom severity reduction and improvement in exercise capacity) and a sustained reduction in sympathetic overdrive to be achieved [17]. Because the sympathetic nervous system has also important close inter-relationships with the inflammatory process and the immune system [18], the observation that physical training may trigger anti-inflammatory effects at the level of skeletal muscle [19] has strengthened the hypothesis that sympathetic neural factors may improve vascular distensibility and arterial compliance in CHF by modulating the inflammatory process [20].

Continuous positive airway pressure has been shown to improve, after months of treatment, flow-mediated vasodilation as an index of endothelial function in patients with documented obstructive sleep apnoea [21]. Although no “ad hoc” study examined the vascular effects of the procedure in CHF complicated by sleep apnoea, it is likely that the improvement in endothelial function described above may also be detectable in this condition. Clear-cut sympathoinhibitory effects, documented as the reduction in venous plasma norepinephrine, norepinephrine spillover and sympathetic nerve traffic, have been reported during long-term treatment with continuous positive airway pressure [16,22,23,24], which thus combines favourable effects on neuroadrenergic and vascular dysfunctions. 

Finally, information on the vascular/sympathetic effects of extracorporeal ultrafiltration in CHF are under evaluation at present. Preliminary observations suggest that the procedure may correct fluid overload typical in CHF with a variety of mechanisms, including the reduction in adrenergic cardiovascular overdrive [25].

## 4. Pharmacological Interventions

Drugs employed in the treatment of CHF display important effects on vascular and sympathetic alterations. They will be here described according to the different classes of compounds.

### 4.1. Beta-Blockers

According to guidelines, beta-adrenergic blocking drugs represent important cornerstones of the therapeutic approaches to CHF, as they improve cardiac haemodynamics and patient survival and reduce cardiovascular complications, including the arrhythmogenic ones [26]. The third generation of beta-blocking agents has clear-cut effects on vascular function, e.g., amelioration of endothelial function and oxidative stress, vascular remodelling and reduction in arterial stiffness [27]. This is specifically the case for carvedilol, bisoprolol and nebivolol, which combine the ability to exert the mentioned favourable effects on vascular function with sympathomodulating properties, as documented by data collected using different methodological approaches to assess sympathetic function, namely, norepinephrine spillover, clinical microneurography and metaiodibenzylguanidine scintigraphic neuroimaging techniques [16]. These results are in sharp contrast with those obtained using second-generation beta-adrenergic blocking drugs such as metoprolol, which failed to demonstrate the ability to exert inhibitory effects on neuroadrenergic function and to improve endothelial and, more in general, vascular functions [16,27].

### 4.2. Diuretic Drugs

Consistent evidence collected throughout the years indicates that diuretic agents such as thiazides and loop diuretics may exert unfavourable effects on vascular and endothelial functions, aggravating the already existing functional alterations [28]. These untoward vascular effects are accompanied (and likely triggered) by adverse consequences on neuroadrenergic function, with evidence of a reinforcement of the already detectable sympathetic overdrive [16]. As unfavourable consequences of these untoward effects, diuretic agents may favour the occurrence of cardiac arrhythmias via sympathetic overactivity and plasma electrolyte alterations [16]. They may also favour the development and progression of insulin resistance, a metabolic disarray that can be quite frequently detected in CHF and appears to be related to sympathetic activation in its pathogenesis [16].

### 4.3. ACE-Inhibitors and Angiotensin II Receptor Antagonists

The pharmacological blockade of the renin–angiotensin system with ACE-inhibitors or angiotensin II receptor blockers results in clear-cut favourable effects on vascular and sympathetic functions. As far as vascular function is concerned, the data collected by our group and others have shown that the distensibility and wall thickness of large arteries (carotid artery and aorta) are favourably affected by ACE-inhibitors, as well as angiotensin II receptor blockers [29]. Evidence that therapeutic interventions based on doubling the daily dosage of ACE-inhibitors or adding an angiotensin II receptor blocker to the existing treatment may provide additional favourable effects on endothelial and vascular functions has also been obtained [30]. This appears to be the case in both very mild New York Heart Association (NYHA) class I-II CHF and more advanced NYHA class III-IV CHF. The favourable vascular and endothelial effects of drugs acting on the renin–angiotensin system in CHF depend on a large variety of mechanisms [16,31], such as (1) the reduction in the circulating plasma levels of soluble endothelial adhesion molecules (VCAM-1), (2) the influence of cyclo-oxygenase-dependent vasoconstricting factors, (3) the blockade of bradykinin degradation and (4) the enhanced synthesis of prostaglandins together with augmented release of nitric oxide by endothelial cells. An additional mechanism, however, is represented by the sympathoinhibitory effects exerted by these compounds, which have been shown to reduce, in CHF patients, cardiac and systemic norepinephrine spillover, the heart-to-mediastinum count ratio with 123-I-metaiodobenzyl guanidine or thallium 201 myocardial scintigraphy and the microneurographic recording of efferent postganglionic sympathetic nerve traffic [7,16,31,32,33,34]. Evidence that the mechanisms of the sympathoinhibitory effects of ACE-inhibitors and angiotensin II receptor blockers, which can be documented in patients with preserved or reduced left ventricular ejection fraction, are multiple has been reported. They indeed include (a) an improvement in baroreflex function, whose physiological tonic restraint on adrenergic drive is markedly impaired in CHF [7] and (b) the interference of the drugs with angiotensin II, which is known to exert sympathoexcitatory effects on both central nervous system and peripheral nervous system [7,16].

Recently, the pharmacological combination of angiotensin II receptor blocker valsartan and neprylisin inhibitor sacubitril (ARNi) has been shown, in the ”Prospective Comparison of ARNi with ARB Global Outcomes in Heart Failure with Preserved Ejection Fraction” (PARAGON-HF) trial, to reduce the mortality rate and hospitalization in mild-to-moderate CHF to a greater extent than the one detected with the angiotensin II receptor blocker alone [35]. Along with the augmentation of the vasodilatatory natriuretic peptides in conjunction with the renin–angiotensin system pharmacological blockade, the drug has been shown to exert neuroadrenergic inhibitory effects, as documented by the reduction (about 30% in magnitude) in the sympathetic nerve traffic values after a two-month treatment in CHF patients with reduced ejection fraction [36]. It is likely that the above-mentioned increase in the plasma levels of natriuretic peptides, together with the sympathoinhibitory effects exerted by the drug, is responsible for the improvement in endothelial function and the reduction in arterial stiffness recently reported in CHF during long-term treatment with ARNi [37,38].

### 4.4. Antialdosterone Drugs

Spironolactone and eplerenone, when added to the existing pharmacological treatment of CHF, counteract the adverse effects of aldosterone on cardiac and vascular functions not only by improving cardiac haemodynamics but also by ameliorating endothelial and vascular functions. This was shown in small studies in which spironolactone short-term administration exerted beneficial effects on endothelial function [39]. On the other hand, in CHF patients, eplerenone administration produced facilitatory effects on the number of circulating endothelial progenitor cells, which are known to exert protective properties on re-endothelialisation and vascular repair [40]. Part of these outcomes, which are responsible for the positive clinical effects of the drugs on patient survival, are likely to be mediated by a reduction in neuroadrenergic cardiovascular drive. In CHF, the sympathoinhibitory effects of spironolactone, when administered alone or on top of conventional pharmacological treatment, have been documented using the scintigraphic neuroimaging technique, showing a substantial reduction in myocardial sympathetic neural drive [41,42].

### 4.5. Old and New Drugs

Old and new pharmacological compounds have been employed throughout the years in the treatment of CHF. A time-honoured approach was based on digitalis compound administration, which allowed favourable effects on cardiac haemodynamics to be obtained. The drug, however, did not reduce the CHF-related mortality and hospitalization rate, becoming, throughout the years and in recent guidelines, a compound with selective indication in CHF, as it can only be used in patients with atrial fibrillation for controlling the ventricular rate despite the use of beta-blockers [26]. The drug has been shown to exert, in CHF, sympathoinhibitory effects, together with an improvement in the baroreceptor control of vagal and neuroadrenergic functions [43]. In contrast, information on the vascular and endothelial effects of digoxin in CHF are lacking, and the only available data refer to healthy subjects, in which drug administration leaves endothelium-dependent vasodilation unaltered [44].

Central sympatholytic agents, such as moxonidine and clonidine, have been employed in CHF due their ability to exert central nervous system inhibition of adrenergic overdrive. These drugs do not display, however, favourable vascular effects, since they enhance CHF-related vasoconstriction [7]. In clinical settings, they have been shown not to be associated with a favourable impact on survival. This is particularly the case for moxonide, whose administration at very high daily dosage (five-to-six times greater than the recommended dose) in the “Moxonidine Congestive Heart Failure” (MOXCON) trial [45] has been shown to increase the already elevated mortality rate.

Another drug class frequently used in clinical practice to reduce total cardiovascular risk, for which evidence showing an improvement, during short-term treatment, in endothelium-dependent vasodilation in CHF patients has been accumulated, is represented by statins. Along with their vasodilating properties, statins have been reported to exert clear-cut sympathoinhibitory effects in CHF patients and may thus also contribute to modulating adrenergic overdrive to the heart and peripheral circulation [46,47].

During recent years, clinical studies have assessed the effects of the drug ivabradine, which acts by inhibiting funny current (If) at the level of the sinoatrial node. By producing a reduction in the heart rate, the drug may decrease the hospitalization rate or cardiovascular death in symptomatic CHF patients [48]. The compound slows down the elevated heart rate typical of advanced CHF with mechanisms largely independent of the adrenergic nervous system [48]. However, an improvement in endothelial function at the level of the microcirculation regimen has been reported during a 4-month treatment [49], a finding that may indicate that in some clinical conditions, the vascular effects of a given therapeutic regimen are independent of the sympathetic ones. Finally, very recently, sodium glucose cotransport protein 2 inhibitors (SGT2i), also known as gliflozins, have also been shown to exert, along with favourable effects on glycaemic control in diabetic patients, promising cardiovascular responses, particularly in CHF patients with reduced or preserved ejection fraction [26,50]. These include a reduction in the hospitalization rate and cardiovascular mortality. They also include an improvement in the renal outcomes and a significant reduction in the circulating plasma markers of decongestion, such as N-terminal pro-B-type natriuretic peptides [51]. Some of these effects may have, as co-mediators, the improvement in endothelial function and the concomitant sympathetic inhibition [52,53].

## 5. Device-Based Interventions

Cardiac resynchronization therapy in heart failure exerts not only an improvement in the clinical symptoms and cardiac haemodynamics but also a decrease in arterial stiffness and a concomitant reduction in sympathetic cardiovascular drive [6,54]. Over the years, other device-based interventions have been promoted in CHF. This is the case for carotid baroreceptor stimulation using a device positioned at the level of the right carotid artery, capable of intermittently stimulating carotid baroreceptors, thus achieving the baroreflex-mediated inhibition of adrenergic drive [55]. The procedure has been initially tested in resistant hypertension, showing the ability to achieve, via the inhibition of sympathetic drive, a significant reduction in ambulatory and clinic blood pressure values. More recently, our group and others have tested the approach in CHF patients with reduced ejection fraction [7,56]. The results can be summarized as follows: First, carotid baroreceptor stimulation improved exercise tolerance, functional class and walking test in CHF patients in NYHA classes III and IV. In addition, 3 months after implantation, the carotid baroreceptor control of adrenergic drive was improved, and this was associated with a reduction in sympathetic neural drive of nearly 30% [56]. By prolonging the follow-up to more than 3 years, it was possible to demonstrate the sustained sympathoinhibitory effects of the procedure, which allows sympathetic activity to be restored to values almost superimposable to those seen in age-matched healthy controls [7]. Despite the marked sympathoinhibitory effects, carotid baroreceptor stimulation in CHF at the 3-month follow-up did not affect arterial stiffness, a result which once more emphasizes the possibility that the behaviour of vascular and endothelial functions does not necessarily go hand-by-hand with that of sympathetic function [7].

Finally, a further approach that has been employed in the treatment of CHF is bilateral renal nerve ablation. The procedure, which, in resistant hypertensive patients, is capable of reducing blood pressure, thus inhibiting the sympathetic overactivity typical of the disease [57], is still under investigation in CHF. At present, no data on sympathetic responses to the procedure in CHF are available, while some evidence suggests the lack of any favourable vascular or endothelial effect in the same pathological state [58].

## 6. Unmet Goals of Therapeutic Interventions and Conclusions

**Figure 2:**
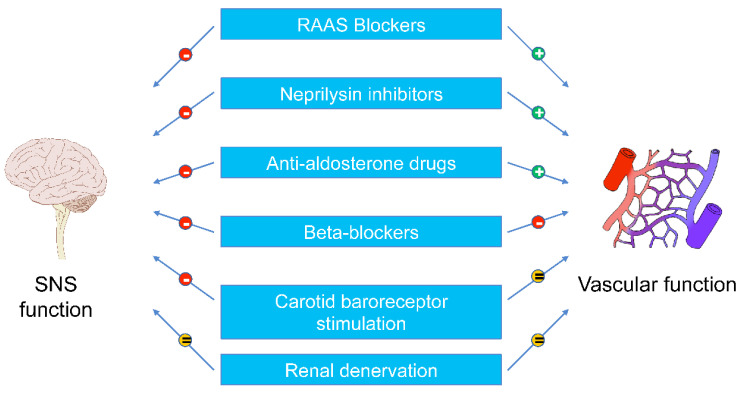
Effects of various therapeutic procedures on sympathetic and vascular dysfunctions in CHF. Symbol “+” means positive effects, and symbol “−” negative effects. RAAS, renin–angiotensin–aldosterone system. Other abbreviations as in Figure 1.

Figure 2 schematically depicts the main results on the sympathetic and vascular effects of pharmacological and non-pharmacological interventions in heart failure treatment described in the present paper. Some goals in therapeutic interventions appear to be still unmet. For example, with very few exceptions, pharmacological, non-pharmacological and device-based interventions, although capable of reducing sympathetic activation and improving vascular function, fail to achieve the full normalization of the different variables. This may explain the finding that therapeutic interventions in CHF fail to normalize cardiovascular risk, allowing the so-called residual risk to persist [59]. The latter may be responsible, for example, for the finding that despite treatment, CHF patients are exposed to an elevated risk of life-threatening cardiac arrhythmias, progression of the disease and fatal complications. A further element of uncertainty in the evaluation of the therapeutic interventions refers to the fact that there is quite large interindividual variability in the sympathetic and vascular responses to a given pharmacological or non-pharmacological intervention, which is very often extremely difficult to predict in current clinical practice.

Both above-mentioned unmet goals in CHF treatment should became important objectives of cardiovascular research in this area to allow the clinical impact of therapeutic interventions and the long-term prognosis of the disease to be improved.

## Data Availability

Not applicable.
